# A Terpyridine Based 1,2,3‐Triazol‐1,4‐diyl‐Fluoroionophore‐A Fluorometric Study Towards 3d Metal Ions in Acetonitrile

**DOI:** 10.1002/open.202400403

**Published:** 2024-12-19

**Authors:** Thomas Schwarze, Holger Müller, Eric Sperlich, Alexandra Kelling, Hans‐Jürgen Holdt

**Affiliations:** ^1^ Institut für Chemie Anorganische Chemie Universität Potsdam Karl-Liebknecht-Str. 24–25 14476 Golm Germany

**Keywords:** Metal ions, Fluorescence, Electron transfer, Charge transfer, Click chemistry

## Abstract

In this paper, we report on the sensing role of the 1,2,3‐triazol unit in a 1,4‐diyl arrangement in a fully π‐conjugated fluorescent probe **1** (cf. Scheme 1) towards the fluorometric detection of 3d metal ions. The 1,2,3‐triazol‐1,4‐diyl‐fluoroionophore **1** was designed in a donor(D)‐acceptor(A) arrangement with a 1,2,3‐triazol unit as a π‐linker between a terpyridine (A) ionophore and a diethylaminocoumarin (D) fluorophore to study the fluorescence behavior towards the divalent 3d metal ions Mn^2+^, Fe^2+^, Co^2+^, Ni^2+^, Cu^2+^ and Zn^2+^. This fluoroionophore **1** is based on an intramolecular charge transfer (ICT) and shows a moderate quantum yield (*φ*_f_) of 0.508 in acetonitrile. A modulation of the ICT process in **1** through Fe^2+^, Co^2+^, Cu^2+^, Ni^2+^ and Zn^2+^ leads to a small red shift of the lowest energetically lying absorption UV/Vis absorption band and to a very strong 3d metal ion induced fluorescence quenching. It should be considered, that the installation of a 1,2,3‐triazole unit as a fully π‐linker in ICT probes originates no ratiometric fluorescence response towards Fe^2+^, Co^2+^, Cu^2+^, Ni^2+^ and Zn^2+^.

## Graphical abstract

We synthesized the 1,2,3‐triazol‐1,4‐diyl‐fluoroionophore **1** in a donor(D)‐acceptor(A) arrangement with a 1,2,3‐triazol unit as a π‐linker between a terpyridine (A) ionophore and a diethylaminocoumarin (D) fluorophore to study the fluorescence behavior of **1** towards the divalent 3d metal ions Mn^2+^, Fe^2+^, Co^2+^, Ni^2+^, Cu^2+^ and Zn^2+^.

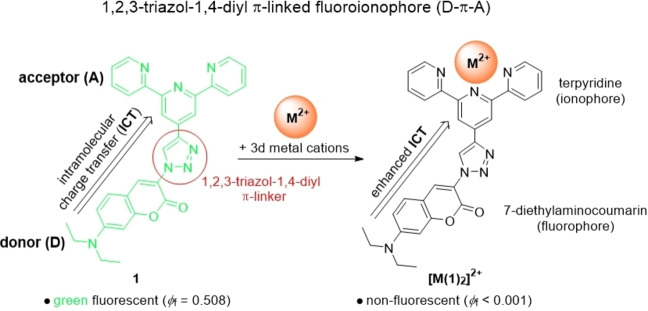

## Introduction

Over the years, fluorescent probes were widely used to detect analytes such as cations in vivo.^[1a,b]^ These probes so called fluoroionophores consist of an ionophore, a cation binding moiety and of a fluorophore, a fluorescence signaling unit. In general, two major design principles were favored to construct fluoroionophores. On the one hand a spacer between the ionophore and fluorophore electronically decouples the two modules from each other. In this fluorophore‐spacer‐ionophore format often a cation induced off switching of a photoinduced electron transfer (PET) process is observed, which leads normally to fluorescence intensity enhancements at a single emission wavelength.^[2a–d]^ On the other hand, fluoroionophores without a spacer in an ionophore π‐conjugated fluorophore arrangement show cation‐induced spectral shifts of their wavelengths and small intensity changes, caused by the modulation of an intramolecular charge transfer (ICT).^[3]^

**Scheme 1:**
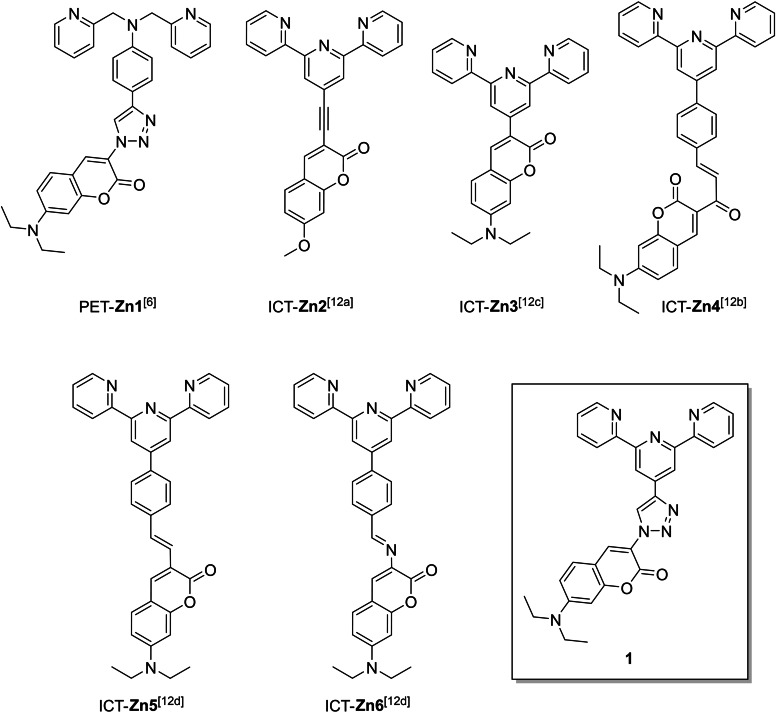
Zn^2+^ responsive fluorescent probes **Zn1**, **Zn2**, **Zn3**, **Zn4**, **Zn5** and **Zn6** as well as studied fluorescent probe **1** (this work).

Recently, we decided to connect directly anilino ionophores with coumarin fluorophores by a π‐linked 1,2,3‐triazole unit which is introduced by a Cu(I)‐catalyzed azide alkyne cycloaddition (CuAAC)^[4a,b]^ reaction. In these 1,2,3‐triazol‐1,4‐diyl‐fluoroionophores for K^+^,^[5a–d]^ Na^+^,^[5e,f]^ Ca^2+^,^[5g]^ Mg^2+[5g]^ or Zn^2+[5g]^ a photo‐induced electron transfer (PET) from the aniline donor to the coumarin acceptor across the virtual triazole spacer takes place and quenches the fluorescence.^[5a–g]^ After addition of the corresponding cation the PET is interrupted, and a fluorescence intensity enhancement (FE) is observed in water. Overall, these 1,2,3‐triazol‐1,4‐diyl‐fluoroionophores for K^+^, Na^+^, Ca^2+^, Mg^2+^ or Zn^2+^ consist of the same PET motif an aniline‐1,2,3‐triazol‐1,4‐diyl‐coumarin arrangement.^[5a–g]^ Further, Zhu et al. reported about a fluorescent probe for Zn^2+^ which is based on this PET motif (cf. Scheme 1, **Zn1**).^[6]^

Further, fluorescent probes which incorporate a 1,2,3‐triazole unit as a spacer or are part of the π‐system of the ionophore or fluorophore were widely used but were the 1,2,3‐triazole acts directly as a π‐bridge between a fluorophore and ionophore were rarely explored.^[7a,b]^ In a systematic study, we investigated the sensing role of the 1,2,3‐triazol unit as a π‐linker in fluoroionophores, which operate by different photophysical principles such as PET, ICT and of a locally excited (LE)/CT state reversal.^[8]^ Further, the LE/CT state reversal probes in a donor‐π‐acceptor (D‐π‐A) arrangement enables the development of ratiometric fluorescent probes for monovalent cations K^+[9a]^ and Na^+^.^[9b]^ Moreover, for divalent cations, such as Zn^2+^, ICT based fluorescent probes were favored because of their ability to induce stronger spectral shifts than monovalent cations.^3^, ^10^ Overall, a benefit of ratiometric metal ion detection in vivo is that this procedure reduces or eliminates distortions caused by photobleaching effects, indicator concentration changes or illumination stabilities.

**Scheme 2:**
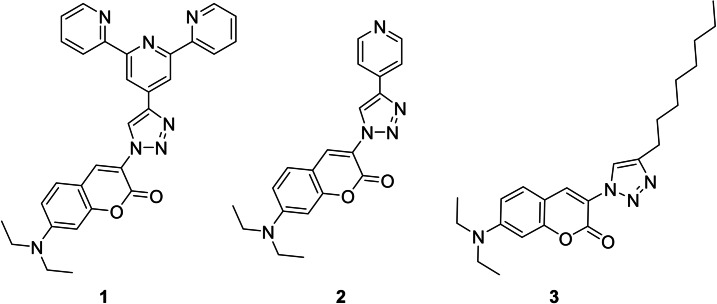
Studied cation‐responsive fluorescent probe **1** and reference compounds **2** and **3**.

In this paper, we wanted to further investigate the sensing function of the 1,2,3‐triazole unit in fluorescent probes. Moreover, we strive to clarify the influence of the structural motif 1,2,3‐triazole in combination with an ICT coumarin fluorophore and its consequences for ratiometric sensing of divalent cations. In general, dialkylamino substituted coumarin derivatives are well known ICT fluorophores.^[11a]^ After excitation of these coumarins two different excited states were mainly observed. A highly fluorescent ICT state and a nonfluorescent twisted ICT state.^[11a]^ The emissive ICT state is very planar and the dialkylated amino group acts as an electron donor (D) and the carbonyl coumarin unit acts as an electron acceptor (A). A manipulation of the push‐pull effect by introducing electron withdrawing groups in the 3‐position results in a bathochromic shift.^[11a]^ Further, to get a fluorescent probe, which shows cation induced wavelength shifts and ideally a ratiometric fluorescence behavior, we designed the ICT fluoroionophore **1** (cf. Scheme 2). We built up fluorescent probe **1** in a donor‐π‐acceptor (D‐π‐A) arrangement, where the terpyridine ionophore (A) and the 1,2,3 triazole unit is part of the π‐system of the coumarin ICT fluorophore (D). We selected as an ionophore a 2,2′:6′,2′′‐terpyridine moiety because the binding characteristics to divalent 3d metal ions are well studied. Further, the terpyridine ionophore is also well investigated in combination with the ICT fluorophore naphthalimide.^[11b]^

Particularly, fluorescent probes based on a terpyridine ionophore in combination with a coumarin fluorophore show a high Zn^2+^ selectivity in H_2_O/DMSO mixtures (cf. **Zn2** and **Zn4**).^[12a,b]^ Further, some Zn^2+^ responsive and ratiometric ICT probes (cf. **Zn2**,^[12a]^ **Zn3**,^[12c]^ **Zn4**,^[12b]^ **Zn5** ^[12d]^ and **Zn6** ^[12d]^) are shown in Scheme 1 consisting of different π‐linkers between the ionophore (terpyridine) and fluorophore (coumarin) such as a double (cf. **Zn5**) or a triple (cf. **Zn2**) bond as well as an enone (cf. **Zn4**) or imine (cf. **Zn6**) group. As rare examples, these ICT probes show a Zn^2+^‐induced red shifting of their fluorescence emissions. A more common strategy to detect ratiometrically Zn^2+^ is to design ICT probes, where Zn^2+^ interacts with the ICT donor (ionophore), which causes a blue shift.^1^, ^13^ Moreover, we synthesized the terpyridine free reference dyes **2** and **3** (cf. Scheme 2).

## Results and Discussion

### Synthesis and Characterization of Fluorescent Dyes 1, 2 and 3

The synthesis of **1**, **2** and **3** (cf. Scheme 2) were realized by CuAAC reactions of commercially available alkynyl functionalized compounds with 3‐azido‐7‐diethylaminocoumarin,^[14]^ respectively.^[15]^ The fluorescent probes **1**, **2** and **3** were characterized by ^1^H‐NMR‐, ^13^C‐NMR‐ spectroscopy and mass spectrometry.^[15]^

**Figure 1:**
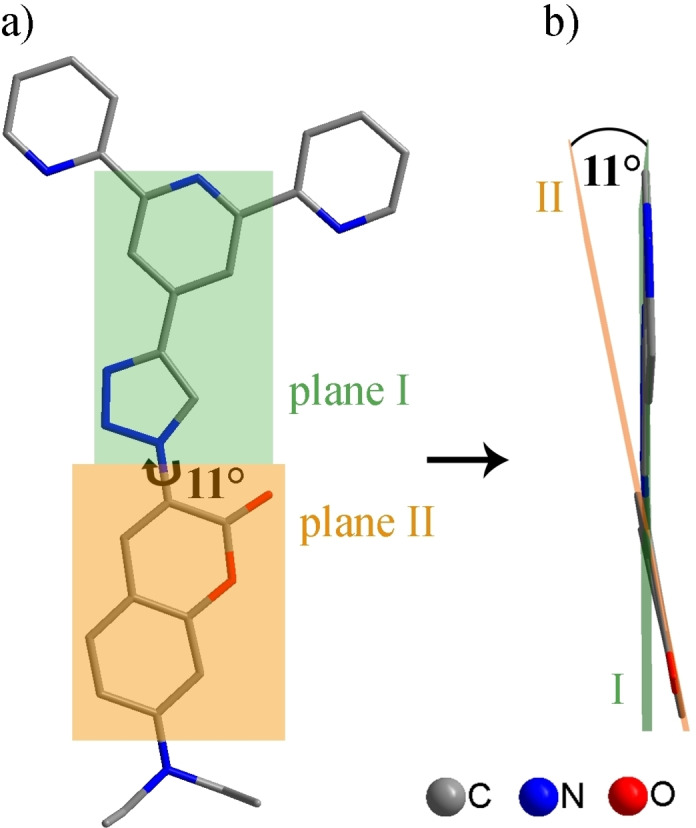
X‐ray structure of **1** a) plane I through the 1,2,3‐triazol‐pyridine moiety (green) and plane II through the coumarin unit (orange). b) A torsion around 90° shows an angle between plane I and plane II of 11°.

Further, the molecular structure of **1** was confirmed by X‐ray analysis. Figure 1 shows the molecular structure of **1** and illustrates that the 1,2,3‐triazol‐terpyridine moiety is nearly coplanar to the coumarin unit (11°) within **1**. The planarity in **1** indicates a nearly full conjugated π‐electron system between the pyridine acceptor (A) and the ICT aminocoumarin donor (D).

### UV/Vis Absorption Studies of 1, 2 and 3 as well as in the Presence of Selected 3d Metal Ions

**Figure 2:**
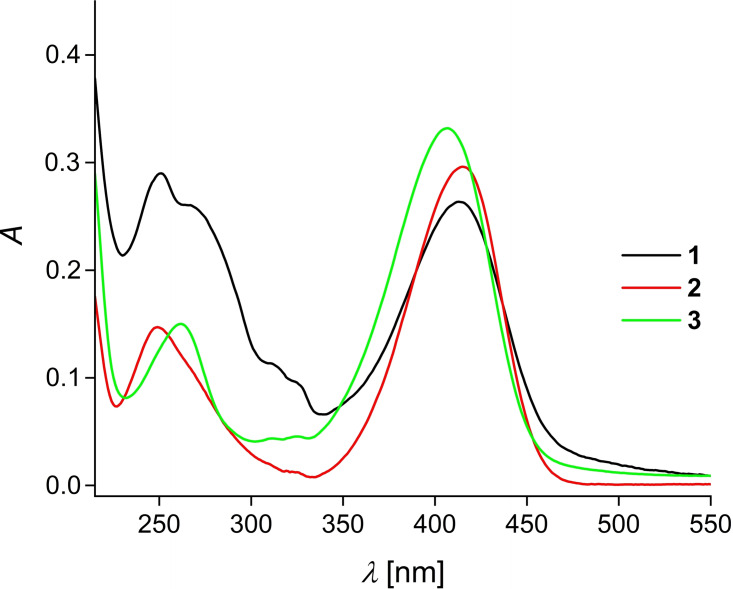
UV/Vis absorption spectra of **1**, **2** and **3** (*c*=10^−5^ M) in acetonitrile.

The UV/Vis absorption spectra of **1**, **2** and **3** in CH_3_CN were displayed in Figure 2. The dyes **1**, **2** and **3** show a very similar shaped long wavelength absorption band peaked for **1** and **2** at 415 nm and for **3** at 407 nm assigning to the coumarinic ICT absorption.^[11a]^ The red shifted absorption of this coumarinic band compared to 7‐diethylaminocoumarin (380 nm in methanol^[16]^) is due to the fact that electron withdrawing groups were introduced on the coumarinic 3‐position in **1**, **2** and **3**. Further, we observed for **3** a weaker red shifted *λ*_max_ indicating that the 1,2,3‐triazol‐terpyridine unit in **1** and **2** has a more withdrawing effect than the 1,2,3‐triazole unit in **3**. Thus, the terpyridine acceptor moiety in **1** and **2** is also part of the π‐conjugated electron system of the coumarinic ICT absorption as also mentioned by the X‐ray structure of **1** (vide supra). Further, this indicates a good electronic communication between the terpyridine acceptor and the aminocoumarin donor in **1** and **2**. Furthermore, the shoulder from 320 to 340 nm in the spectrum of **1** can be attributed to a CT from the triazole unit to the terpyridine heterocycle.^[17]^ Moreover, the UV‐Vis absorption spectrum of **1** shows from 220 nm to 270 nm a broad absorption band which can be attributed to a mixture of π‐π* and n‐π* transitions localized on the terpyridine unit.^[18a,b]^

**Figure 3:**
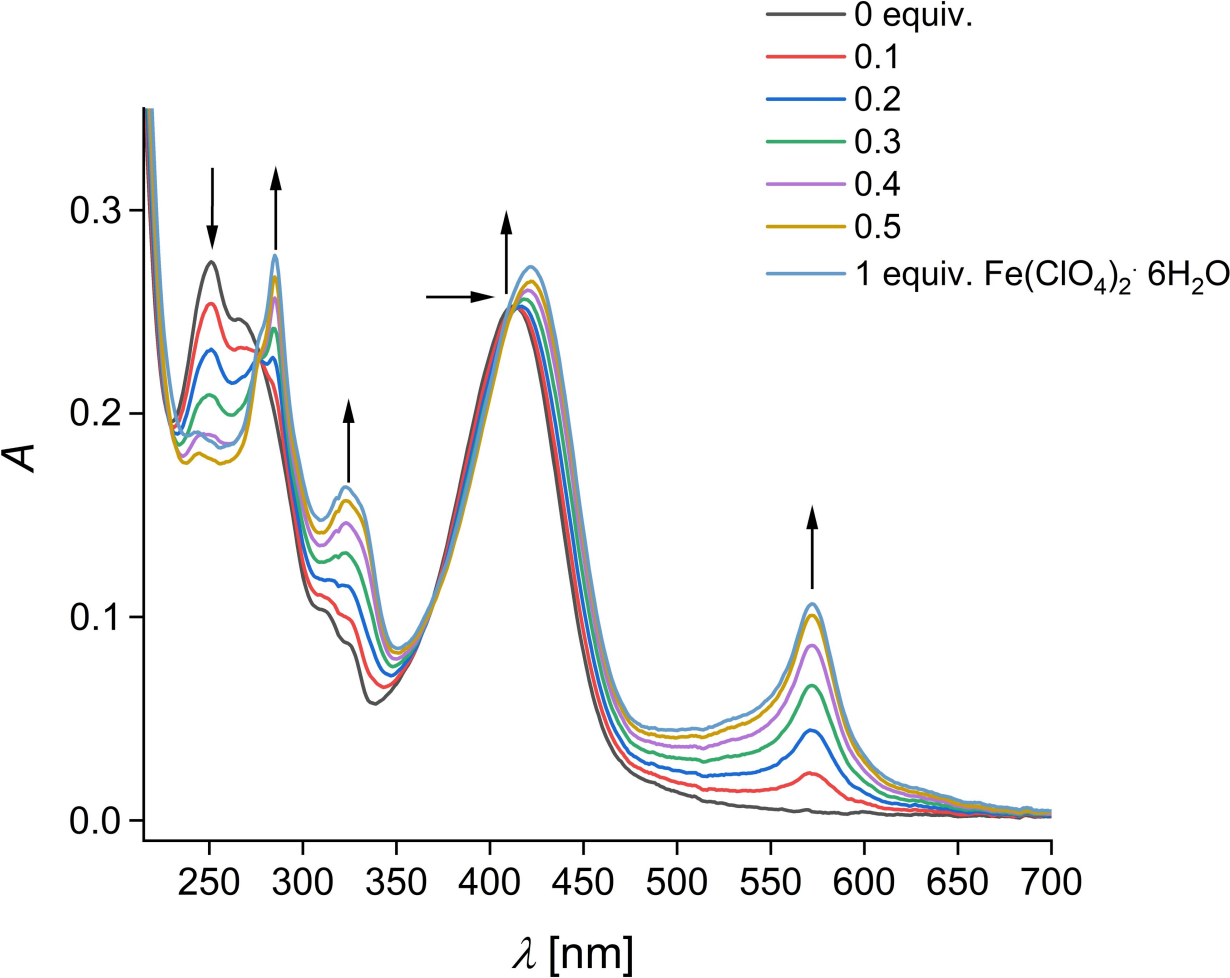
UV/Vis absorption spectra of **1** (*c*=10^−5^ M) in the presence of increasing Fe^2+^ equivalents in acetonitrile.

In a next step, we studied the influence of the 3d metal ions Mn^2+^, Fe^2+^, Co^2+^, Ni^2+^, Cu^2+^ and Zn^2+^ towards the UV/Vis absorption behavior of **1** in acetonitrile (cf. Figures 3 and S5a–f). Figure 3 shows the UV/Vis absorption titration of **1** with iron(II) perchlorate hexahydrate, exemplary for the other titration experiments with the perchlorate salts of Mn^2+^, Co^2+^, Ni^2+^, Cu^2+^ and Zn^2+^. Noticeable, during the titration of **1** with Fe^2+^ a new absorption band, until the addition of 0.5 equivalents, at 570 nm with an extinction coefficient of 1600 M^−1^ cm^−1^ appears (cf. Figure 3) which can be attributed to a metal to ligand charge transfer (MLCT).^[19a,b]^ We also observed for **1**+Co^2+^ (cf. Figure S5c) an increased absorption at around 525 nm with a very small extinction coefficient of 200 M^−1^ cm^−1^ which is also assigned to a MLCT.^[20]^ Commonly, we found for **1** in the presence of Mn^2+^, Fe^2+^, Co^2+^, Ni^2+^, Cu^2+^ and Zn^2+^ only a small and enhanced red shift (approximately 5 nm) of the coumarinic ICT absorption band (cf. Figure S5a–f) caused by the interaction of the divalent cation with the π‐system of the terpyridine acceptor which enhances the electron withdrawing character of this group.^[3]^ The complexation behavior of **1** with these cations can be better seen from the terpyridine absorptions at around 250 nm (cf. Figures 3 and S5a–f). The band at 250 nm is decreased and shifted to 285 nm by the tested cations. The titration curves at 250 nm (cf. Figure S6a) show a good linear decrease for **1**+Mn^2+^, **1**+Fe^2+^, **1**+Cu^2+^ and **1**+Zn^2+^, respectively, with a sharp endpoint at a metal/ligand ratio of 0.5 : 1, indicating a 1 : 2 complex formation in solution (cf. Figure S6a).^21^, ^22^ Further, we also observed for **1**+Co^2+^ and **1**+Ni^2+^ a decrease of the absorption band at 250 nm to a metal/ligand ratio of 0.33 : 1, indicating a 1 : 3 complex formation (cf. Figure S6b). Overall, a calculation of the binding constants for the 1 : 2 complexes of **1** with Mn^2+^, Fe^2+^, Cu^2+^ and Zn^2+^ is difficult due to the lack of curvature in their titration curves^[21]^ but the straight slope and its high saturation in the presence of 0.5 metal(II)salt equivalents indicating high and very similar to each other binding constants as also found for set of terpyridine to metal 2 : 1 complexes.^21^, ^22^ All UV/Vis absorption titrations show at least two isosbestic points assuming that several species are existing during the titration process (cf. Figure S5a–f).

### Fluorescence Studies of 1, 2 and 3 as well as in the Presence of Selected 3d Metal Ions

At first, we recorded fluorescence spectra of **1**, **2** and **3** in acetonitrile. The fluorescent probes **1** and **2** show very similar fluorescence emission maxima (485 nm, cf. Table 1). For **3**, we found a slightly blue shifted emission maximum of 470 nm (cf. Table 1). Overall, the emission maxima of **1**, **2** and **3** were red shifted compared to 7‐diethylaminocoumarin (445 nm in methanol^[16]^) which further indicates that the electron withdrawing fragments in coumarinic 3‐position are part of the π‐conjugated electron system of the coumarinic ICT absorption. The fluorescence quantum yields of **1**, **2** and **3** in acetonitrile were also very similar to each other (0.508 (**1**), 0.650 (**2**) and 0.614 (**3**)), but higher than for 7‐diethylaminocoumarin (*φ*_f_=0.27 in methanol^[16]^). Thus, **1** is a highly fluorescent candidate for a fluorometric investigation towards divalent cations.

**Table 1:** Photophysical properties of **1**, **2** and **3** in acetonitrile.

dye	*λ*_abs_^[a]^ [nm]	*λ*_f(max)_^[b]^ [nm]	*φ*_f_^[c]^
**1**	415, 250	485	0.508
**2**	415, 249	485	0.650
**3**	407, 261	470	0.614

[a] Selected absorptions. [b] Fluorescence maxima *λ*_f(max)_. [c] Fluorescence quantum yields ±15 %.

**Figure 4:**
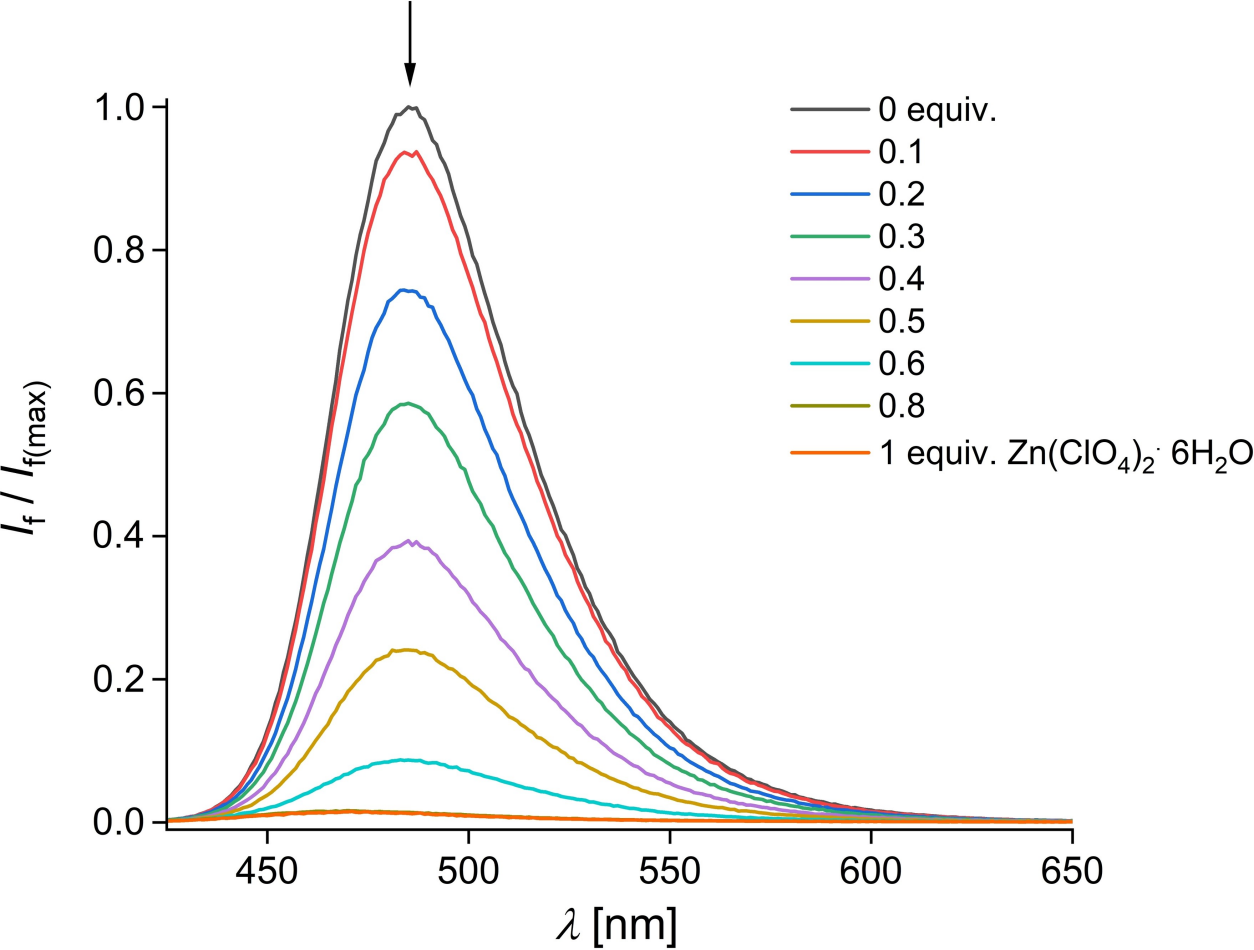
Fluorescence response of **1** (*c*=10^−6^ M, *λ*_ex_=413 nm) in the presence of increasing Zn^2+^ equivalents in acetonitrile.

Moreover, we measured the fluorescence intensity of **1** in the presence of various 3d metal ions such as Mn^2+^, Fe^2+^, Co^2+^, Ni^2+^, Cu^2+^ and Zn^2+^ in acetonitrile. Figure 4 shows the fluorescence intensity changes of **1** in the presence of increasing Zn^2+^ equivalents. For all tested divalent metal ions, we observed a strong fluorescence quenching of **1** (cf. Figure S7a–f). The fluorescence titration curves of **1** at 485 nm (cf. Figure S8a and b) show a very similar behavior as already found by the UV/Vis absorption experiments. Again, for **1**+Mn^2+^, **1**+Fe^2+^, **1**+Cu^2+^ and **1**+Zn^2+^, we found hints for a 1 : 2 complex formation and for **1**+Co^2+^ and **1**+Ni^2+^ we assume a 1 : 3 complex formation in solution (cf. Figure S8b). Furthermore, the quenching effect of Mn^2+^, Fe^2+^, Cu^2+^ and Zn^2+^ to **1** is very similar to each other assuming a comparable binding strength by a non‐following of the Irving‐Williams series.^[23]^ The strong and nearly complete fluorescence quenching of **1** by these tested cations is attributed to many factors, for example their paramagnetic nature, heavy atom effect, a strong binding with the terpyridine unit and much more.^21^, ^22^, ^23^ Interestingly, we found no red shift of the fluorescence emission of **1** in the presence of Zn^2+^, as shown e. g. for the ratiometric fluorescent probes **Zn2** and **Zn4** (cf. Scheme 1). For further comparison of **1** with previously reported fluorescent probes consisting of a terpyridine ionophore and a coumarin fluorophore (**Zn1**–**Zn6**) see Table S2 in supporting information.^[15]^

## Conclusions

We synthesized by a CuAAC reaction the ICT fluoroionophore **1** consisting of a terpyridine ionophore and a coumarin fluorophore in a D‐π‐A arrangement. The 1,2,3‐triazol‐1,4‐diyl‐fluoroionophore **1** (*φ*_f_=0.508) is fluorescent and shows in the presence of 3d metal ions such as Mn^2+^, Fe^2+^, Co^2+^, Ni^2+^, Cu^2+^ and Zn^2+^ a very strong cation induced fluorescence quenching in acetonitrile. Overall, for the construction of ratiometric ICT fluoroionophores for Zn^2+^, the π‐linkage between the ionophore and fluorophore should be carefully chosen, because the 1,2,3‐triazole unit changes the recognition behavior towards Zn^2+^ compared to other well known π‐linked ICT fluoroionophores for Zn^2+^ consisting of a terpyridine ionophore and a coumarin fluorophore (cf. Scheme 1). Currently, we are synthesizing in our lab ratiometric and Zn^2+^‐selective 1,2,3‐triazol‐1,4‐diyl‐fluoroionophores in a donor‐π‐acceptor (D‐π‐A) arrangement based on a LE/CT state reversal sensing mechanism.

## Conflict of Interests

The authors declare no conflict of interest.

## Supporting information

As a service to our authors and readers, this journal provides supporting information supplied by the authors. Such materials are peer reviewed and may be re‐organized for online delivery, but are not copy‐edited or typeset. Technical support issues arising from supporting information (other than missing files) should be addressed to the authors.

Supporting Information

## Data Availability

The data that support the findings of this study are available in the supplementary material of this article.
