# Supplementary material for: A Terpyridine Based 1,2,3‐Triazol‐1,4‐diyl‐Fluoroionophore‐A Fluorometric Study Towards 3d Metal Ions in Acetonitrile
Source: ChemistryOpen. 2024 Dec 19;14(6):e202400403. doi: 10.1002/open.202400403 (PMC13062935; doi:10.1002/open.202400403)
Supplement: Supplementary file 1 — Supporting Information [file OPEN-14-e202400403-s001.pdf]

ChemistryOpen

Supporting Information

A Terpyridine Based 1,2,3-Triazol-1,4-diyl-Fluoroionophore-A Fluorometric Study Towards 3d Metal Ions in Acetonitrile

Thomas Schwarze,* Holger Müller, Eric Sperlich, Alexandra Kelling, and Hans-Jürgen Holdt

Experimental Section

1. General Methods and reagents

All commercially available chemicals were used without further purification. Solvents were distilled prior use. ^{1}H and ^{13}C NMR spectra were recorded on 300 MHz or 400 MHz instruments, respectively. Data are reported as follows: chemical shifts in ppm (δ), multiplicity (s = singlet, brs = broad singlet, d = doublet, t = triplet, dd = doublet of doublets, q = quadruplet, quint = quintuplet, m = multiplet), integration, coupling constant (Hz). ESI spectra were recorded using a Micromass Q-TOF micro mass spectrometer in a positive or negative electrospray mode. Column chromatography was performed with silica gel (Merck; silica gel 60 (0.04-0.063 mesh)).

1.1 Synthetic procedures of the fluorescent probe 1 as well as reference dyes 2 and 3

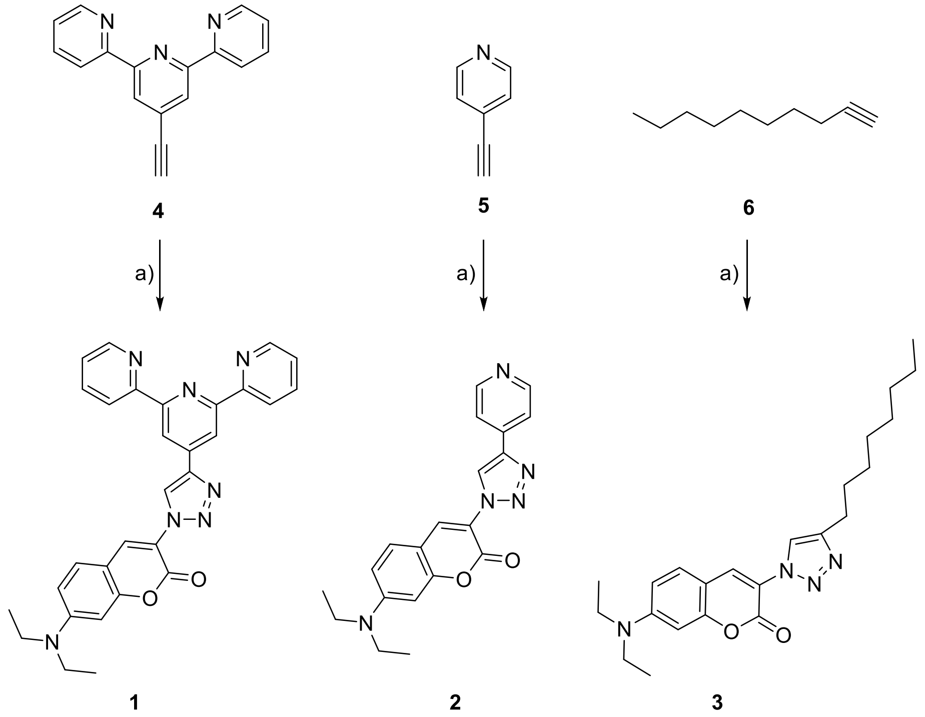

Scheme S1. Synthetic route of the cation-responsive fluorescent probe **1** as well as of reference compounds **2** and **3**: a) 3-azido-7-diethylaminocoumarin[1], $CuSO_4$, Na ascorbate, THF/H_2O, 60 °C.

General CuACC procedure for 1, 2 and 3: A mixture of the corresponding commercially available alkynyl compound **4**, **5** or **6** (0.415 mmol) and 3-azido-7-diethylaminocoumarin[1]

[1] K. Sivakumar, F. Xie, B. M. Cash, S. Long, H. N. Barnhill, Q. Wang, *Org. Lett.* **2004**, *6*, 4603-4606.

(0.415 mmol), $CuSO_4{\cdot}5H_2O$ (5.3 mg) and sodium ascorbate (8.2 mg) in 9 ml THF/H_2O (v/v, 2/1) was stirred at 60 °C for 48 hours. After that 5 mL H_2O were added to the mixture and then extracted with $CHCl_3$ (30 mL). The organic layer was dried with $MgSO_4$ and concentrated in vacuo. The residue was purified by column chromatography on silica using $CHCl_3$/CH_3OH (v/v, 30/1) as an eluent mixture to afford **1** and **2** as yellow solids and **3** as a yellow oil.

Fluorescent probe 1: Yield 96% (205 mg). ^{1}H-NMR ($C_2D_2Cl_4$, 400 MHz): δ = 9.06 (s, 1H), 8.85 (brs, 2H), 8.68 (brs, 2H), 8.59 (brs, 2H), 8.41 (s, 1H), 7.84 (brs, 2H), 7.40 (d, J = 8.9 Hz, 1H), 7.32 (brs, 2H), 6.64 (d, J = 8.9 Hz, 1H), 6.51 (s, 1H), 3.38 (q, J = 6.7 Hz, 4H), 1.17 ppm (t, J = 6.7 Hz, 6H); ^{13}C-NMR (CD_2Cl_2, 75 MHz): δ = 157.11, 156.30, 152.20, 149.60, 145.94, 140.24, 137.24, 135.20, 132.32, 130.44, 128.86, 124.35, 122.66, 121.43, 117.50, 117.04, 110.54, 107.29, 97.30, 45.38, 12.57 ppm; HRMS (ESI): m/z calcd for $C_{30}H_{25}N_7O_2$+H^+: 516.2142 [M+H]$^+$; found: 516.2165.

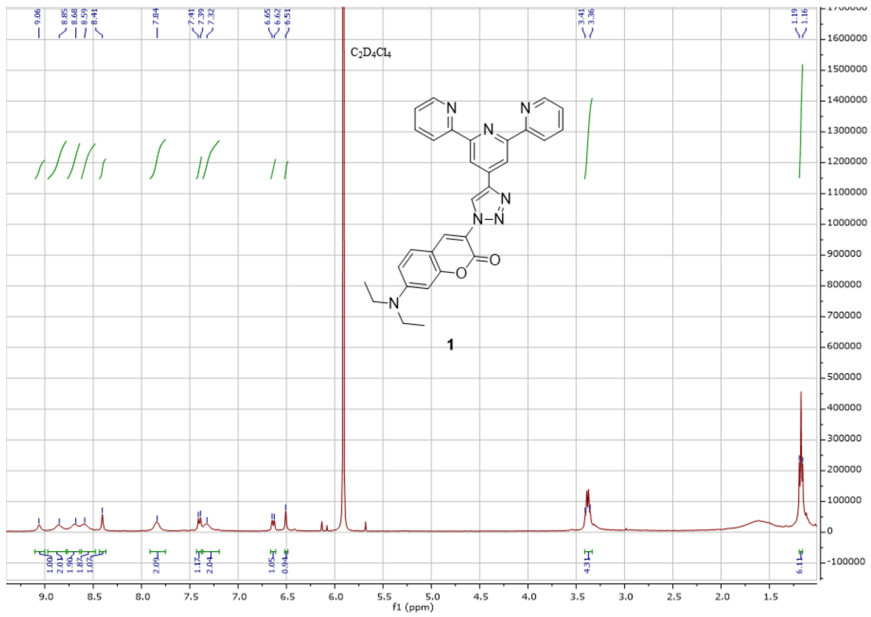

Figure S1. ^{1}H-NMR spectrum of **1** in $C_2D_2Cl_4$.

Fluorescent probe 2: Yield 32% (48 mg); ^{1}H-NMR (C_2D_6OS, 400 MHz): δ = 9.23 (s, 1H), 8.67 (brs, 2H), 8.54 (s, 1H), 7.93 (d, J = 5.1 Hz, 2H), 7.66 (d, J = 9.0 Hz, 1H), 6.85 (dd, J = 9.0 Hz, J = 2.5 Hz, 1H), 6.70 (d, J = 2.4 Hz, 1H), 3.49 (q, J = 6.9 Hz, 4H), 1.16 ppm (t, J = 6.9 Hz, 6H); ^{13}C-NMR (C_2D_6OS, 100 MHz): δ = 166.00, 156.31, 151.21, 150.90, 144.51, 138.05, 131.19, 125.05, 124.69, 121.87, 120.12, 110.62, 106.87, 96.90, 44.73, 12.79 ppm; HRMS (ESI): m/z calcd for $C_{20}H_{19}N_5O_2$+H^+: 362.1611 [M+H]$^+$; found: 362.1866.

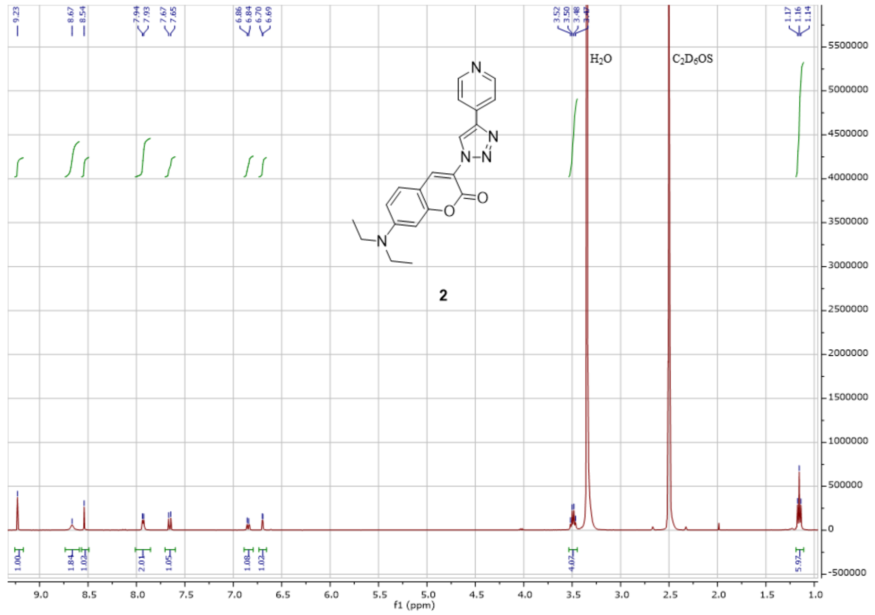

Figure S2. ^{1}H-NMR spectrum of **2** in C_2D_6OS.

Fluorescent probe 3: Yield 74% (122 mg); ^{1}H-NMR ($CDCl_3$, 400 MHz): δ = 8.34 (s, 1H), 8.26 (s, 1H), 7.38 (d, J = 8.8 Hz, 1H), 6.65 (dd, J = 2.5, J = 8.9 Hz, 1H), 6.53 (d, J = 2.4 Hz, 1H), 3.43 (q, J = 7.1 Hz, 4H), 2.76 (t, J = 7.7 Hz, 2H), 1.71 (quint, J = 7.6 Hz, 2H), 1.38-1.17 (m, 16H), 0.86 ppm (t, J = 7.3 Hz, 3H); ^{13}C NMR ($CDCl_3$, 100 MHz): δ = 156.99, 155.62, 151.33, 148.21, 134.33, 129.83, 121.55, 117.16, 109.94, 107.10, 96.95, 44.92, 31.79, 29.31, 29.28, 29.21, 29.15, 25.64, 22.60, 14.04, 12.35 ppm; HRMS (ESI): m/z calcd for $C_{23}H_{32}N_4O_2+H^+$: 397.2598 $[M+H]^+$; found: 397.2558.

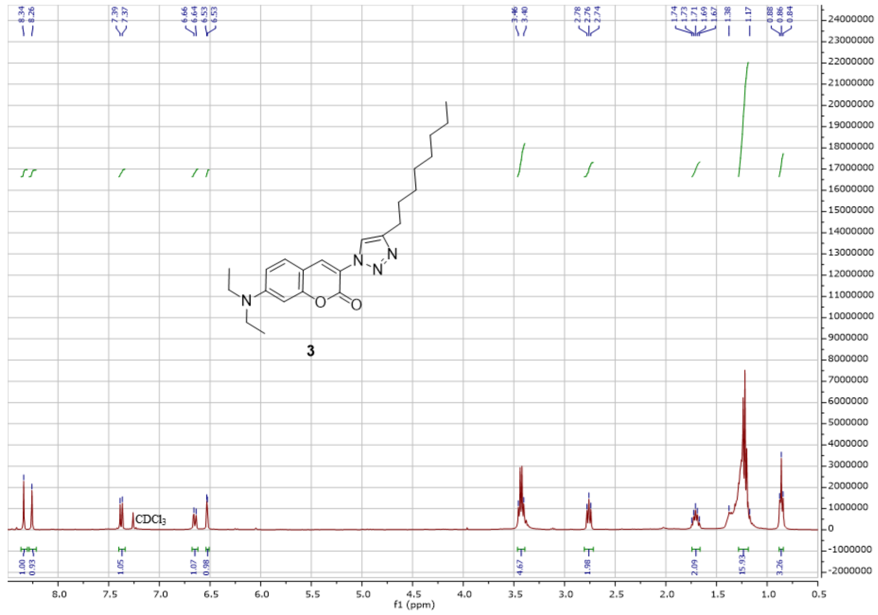

Figure S3. ^{1}H-NMR spectrum of **3** in $CDCl_3$.

2. UV/Vis absorption measurements

UV/Vis absorption measurements were recorded on a Perkin Elmer Lambda 950 spectrophotometer using 1 cm path length quartz cuvettes.

2.1 UV/Vis absorption spectra of 1, 2 and 3 in CH_3CN

The absorption spectra of **1**, **2** and **3** ($c = 10^{-5}$ M) were measured in acetonitrile (cf. Figure S1).

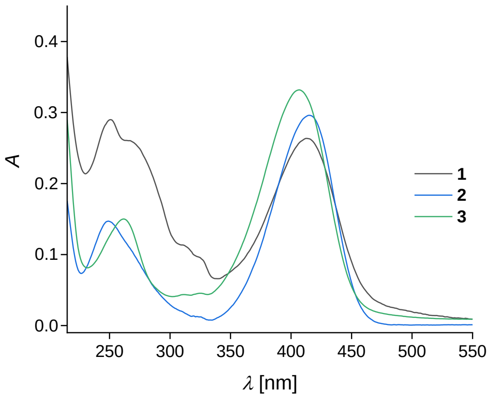

Figure S4. UV/Vis absorption spectra of **1**, **2** and **3** ($c = 10^{-5}$ mol·l^{-1}) in acetonitrile.

2.2 UV/Vis absorption spectra of 1 in the presence of $Mn(ClO_4)_2{\cdot}6H_2O$, $Fe(ClO_4)_2{\cdot}6H_2O$, $Co(ClO_4)_2{\cdot}6H_2O$, $Ni(ClO_4)_2{\cdot}6H_2O$, $Cu(ClO_4)_2{\cdot}6H_2O$ or $Zn(ClO_4)_2{\cdot}6H_2O$ in CH_3CN

UV/Vis titration experiments of **1** ($c = 10^{-5}$ mol·l^{-1}) were carried out by addition of microliter amounts of standard solutions of $Mn(ClO_4)_2{\cdot}6H_2O$, $Fe(ClO_4)_2{\cdot}6H_2O$, $Co(ClO_4)_2{\cdot}6H_2O$, $Ni(ClO_4)_2{\cdot}6H_2O$, $Cu(ClO_4)_2{\cdot}6H_2O$ or $Zn(ClO_4)_2{\cdot}6H_2O$ ($c = 10^{-4}$ mol·l^{-1}) in acetonitrile (cf. Figures S5a-S5f) and between every step a period of 10 minutes were waited. All spectra are concentration corrected.

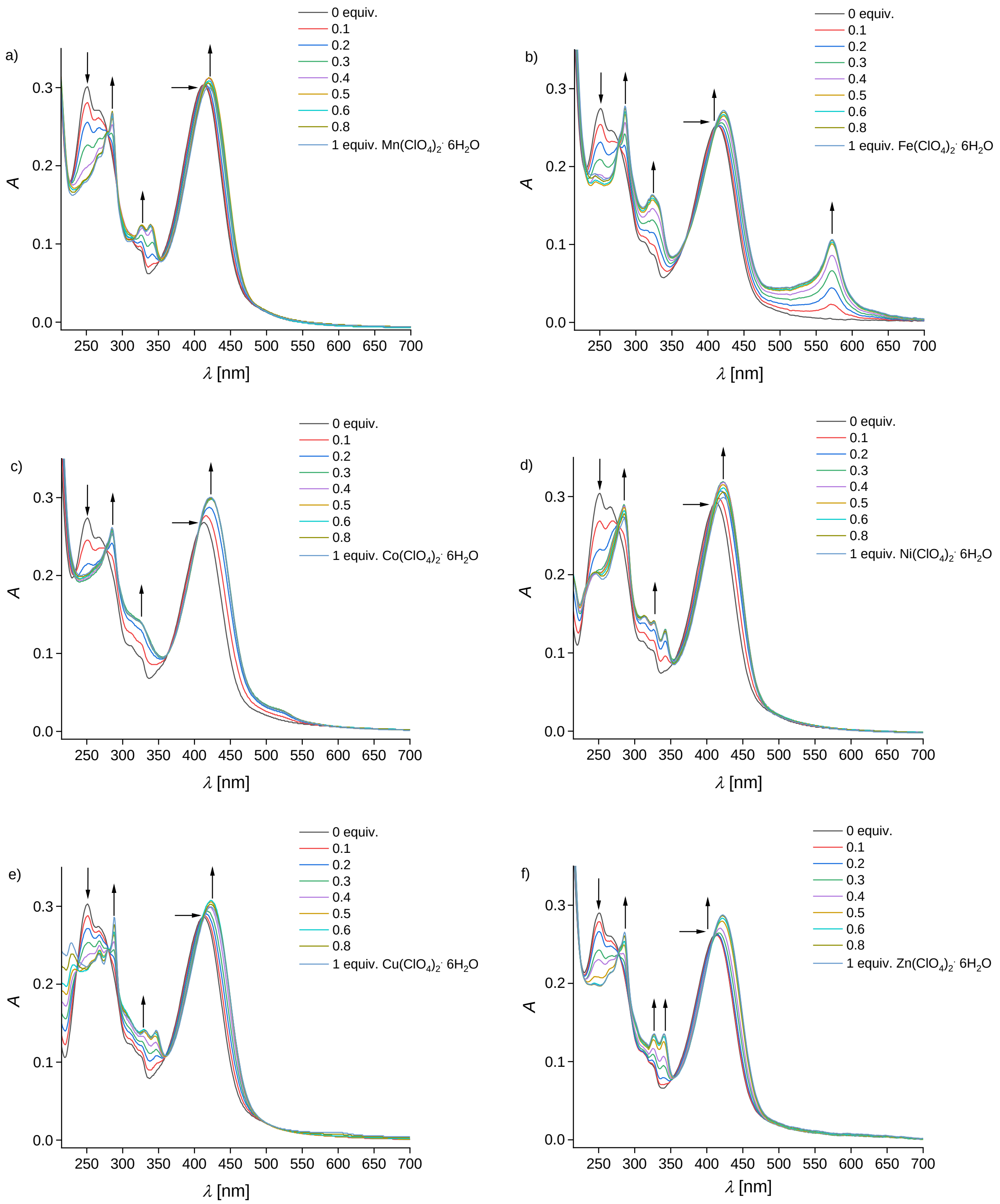

Figure S5. UV/Vis absorption spectra of **1** (c = 10^{-5} mol·l^{-1}) in the presence of increasing equivalents of a) $Mn(ClO_4)_2{\cdot}6H_2O$, b) $Fe(ClO_4)_2{\cdot}6H_2O$, c) $Co(ClO_4)_2{\cdot}6H_2O$, d) $Ni(ClO_4)_2{\cdot}6H_2O$, e) $Cu(ClO_4)_2{\cdot}6H_2O$ and f) $Zn(ClO_4)_2{\cdot}6H_2O$ (c = 10^{-4} mol·l^{-1}) in acetonitrile.

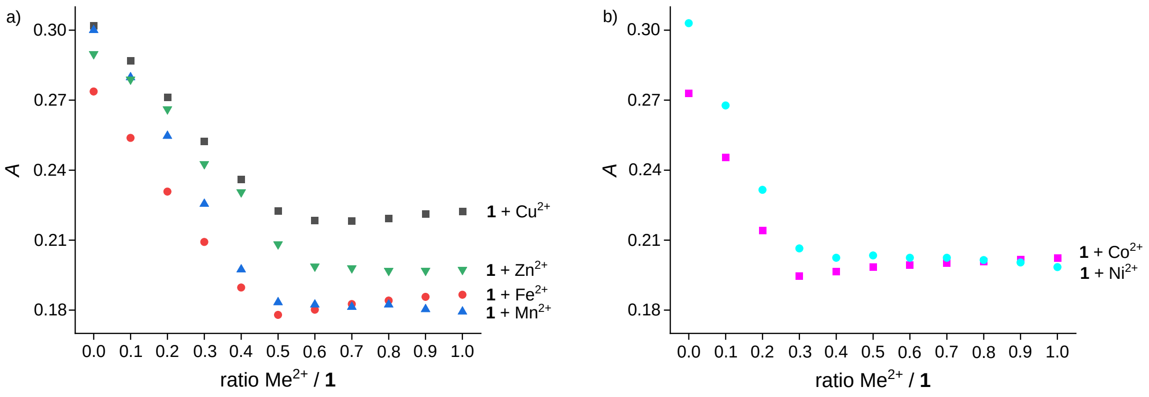

Figure S6. Titration curves of **1** ($c = 10^{-5}$ mol·l^{-1}) in the presence of a) $Mn(ClO_4)_2{\cdot}6H_2O$, $Fe(ClO_4)_2{\cdot}6H_2O$, $Cu(ClO_4)_2{\cdot}6H_2O$ and $Zn(ClO_4)_2{\cdot}6H_2O$ and b) $Co(ClO_4)_2{\cdot}6H_2O$ and $Ni(ClO_4)_2{\cdot}6H_2O$ in acetonitrile at 250 nm.

3. Fluorescence measurements

Fluorescence measurements were carried out with a Fluoromax 3 spectrometer (Horiba Jobin Yvon) using sealed quartz cuvettes. Fluorescence quantum yields were determined using a PL quantum yield measurement system C9920-2 (Hamamatsu, Japan).

3.1 Fluorescence intensity spectra of 1 in the presence $Mn(ClO_4)_2{\cdot}6H_2O$, $Fe(ClO_4)_2{\cdot}6H_2O$, $Co(ClO_4)_2{\cdot}6H_2O$, $Ni(ClO_4)_2{\cdot}6H_2O$, $Cu(ClO_4)_2{\cdot}6H_2O$ or $Zn(ClO_4)_2{\cdot}6H_2O$ in CH_3CN

Fluorescence spectra of **1**, **2** and **3** ($c = 10^{-6}$ M) were measured in acetonitrile.

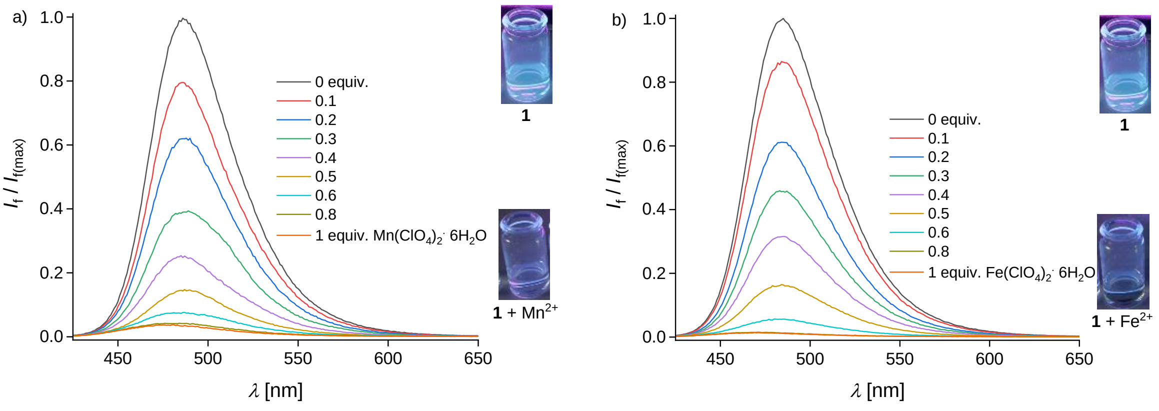

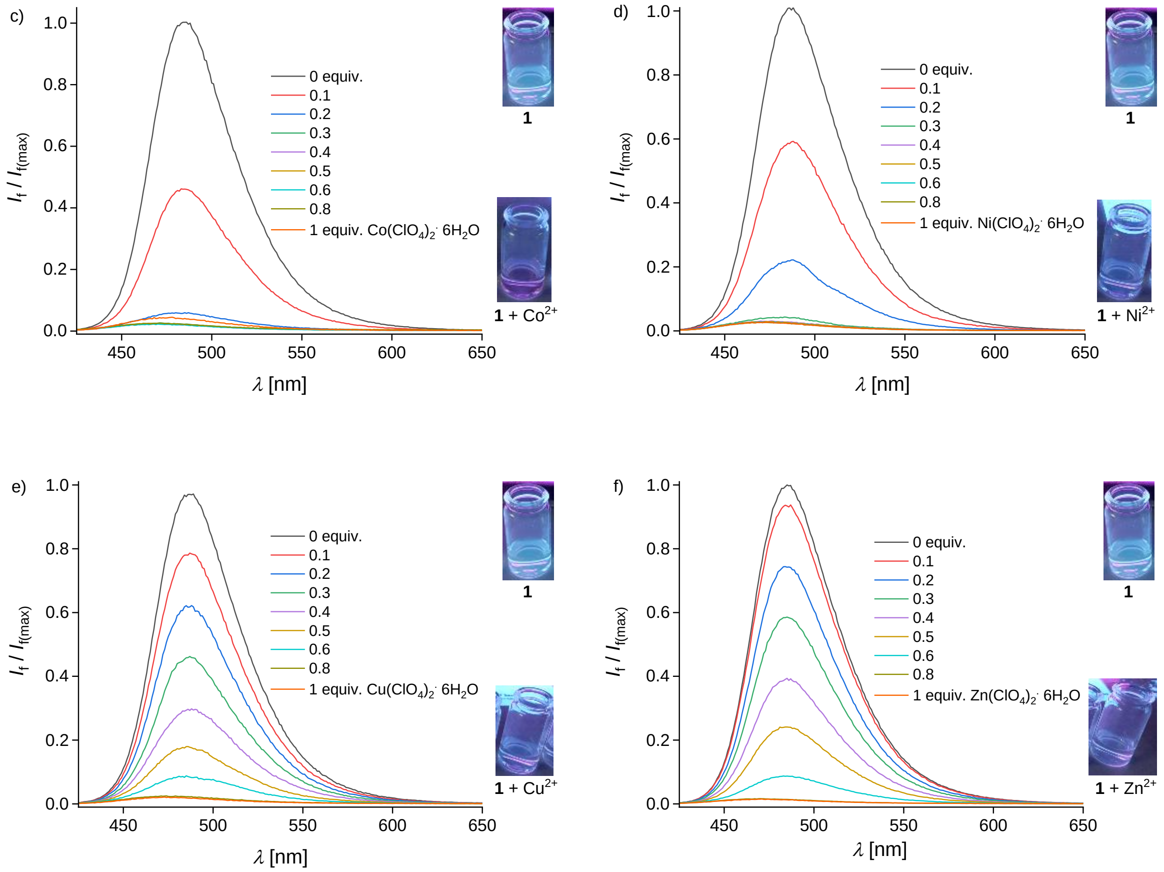

Figure S7. Fluorescence emission spectra of **1** (c = 10^{-6} mol·l^{-1}, λ_{ex} = 413 nm) in the presence of increasing equivalents of a) $Mn(ClO_4)_2{\cdot}6H_2O$, b) $Fe(ClO_4)_2{\cdot}6H_2O$, c) $Co(ClO_4)_2{\cdot}6H_2O$, d) $Ni(ClO_4)_2{\cdot}6H_2O$, e) $Cu(ClO_4)_2{\cdot}6H_2O$ and f) $Zn(ClO_4)_2{\cdot}6H_2O$ (c = 10^{-5} mol·l^{-1}) in acetonitrile. Insets: photographs of fluorescence of **1** and of **1** + cations.

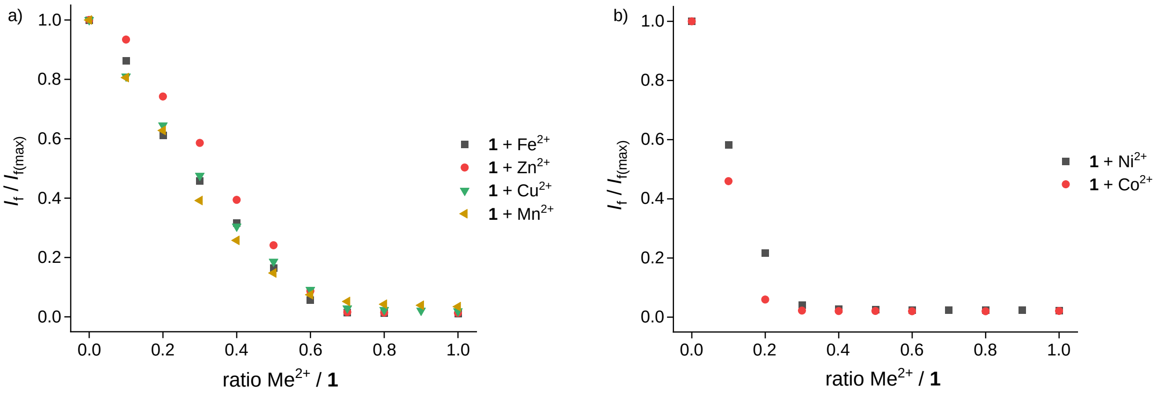

Figure S8. Titration curves of **1** (c = 10^{-5} mol·l^{-1}) in the presence of a) $Mn(ClO_4)_2{\cdot}6H_2O$, $Fe(ClO_4)_2{\cdot}6H_2O$, $Cu(ClO_4)_2{\cdot}6H_2O$ and $Zn(ClO_4)_2{\cdot}6H_2O$ and b) $Co(ClO_4)_2{\cdot}6H_2O$ and $Ni(ClO_4)_2{\cdot}6H_2O$ in acetonitrile at 485 nm.

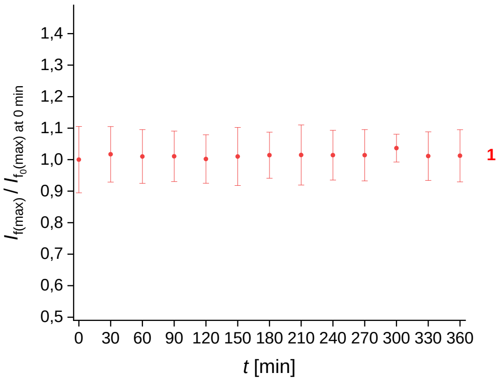

Figure S9. Fluorescence of **1** at 485 nm in acetonitrile ($c = 10^{-6}$ M, $\lambda_{ex} = 413$ nm) which was measured every 30 min by irradiation at 413 nm.

4. General details of X-ray structure analysis

The crystal structures of the compound **1** was determined by single crystal structure analysis. Suitable single crystals were selected using a Leica M205C light microscope and separated with perfluoropolyalkylether oil. X-ray crystal structure analysis was performed on an IPDS-II diffractometer (Stoe) with monochromated Mo-*K*α radiation ($\lambda = 0.71073$ Å). The data correction was performed using the program X-Area.[2] The structure was solved by direct methods and refined against F^2 on all data by full-matrix least-squares using the SHELX suite of programs.[3a,b] All non-hydrogen atoms were refined anisotropically; the hydrogen atoms were placed on calculated positions. The crystal structure was visualized with Diamond 4,[4] **Table 1** was generated using FinalCIF.[5] The data (CCCD 2247272) can be obtained free of charge from The Cambridge Crystallographic Data Centre, http://www.ccdc.cam.ac.uk.

[2] STOE & Cie GmbH, X-Area. software package for collecting single-crystal data on STOE area-detector diffractometers, for image processing, for the correction and scaling of reflection intensities and for outlier rejection, STOE & Cie GmbH, Darmstadt, **2018**.
[3] a) G. M. Sheldrick, *Acta crystallographica. Section A, Foundations of crystallography* **2008**, *64*, 112; b) G. M. Sheldrick, *Acta Cryst C* **2015**, *71*, 3.
[4] K. Brandenburg, H. Putz, *Diamond. Crystal and Molecular Structure Visualization*, Crystal Impact, Bonn, **2020**.
[5] D. Kratzert, *FinalCif.*

4.1 Crystallographic Data

Table 1. Crystal data and details of structure refinement for the compound **1**.

CCDC number	2247272
Empirical formula	$C_{32}H_{27}Cl_6N_7O_2$
Formula weight	754.30
Temperature [K]	210(2)
Crystal system	triclinic
Space group (number)	$P\overline{1}$ (2)
a [Å]	9.870(2)
b [Å]	11.113(2)
c [Å]	16.622(3)
α [°]	72.81(3)
β [°]	72.81(3)
γ [°]	86.29(3)
Volume [$Å^3$]	1663.4(7)
Z	2
ρ_{calc} [gcm^{-3}]	1.506
μ [mm^{-1}]	0.552
F(000)	772
Crystal size [mm^3]	0.600×0.327×0.080
Crystal colour	yellow
Crystal shape	prism
Radiation	Mo K_α (λ=0.71073 Å)
2θ range [°]	3.84 to 58.75 (0.72 Å)
Index ranges	$-13 \leq h \leq 13$ $-15 \leq k \leq 15$ $-22 \leq l \leq 22$
Reflections collected	30721
Independent reflections	8965 R_{int} = 0.0257 R_{sigma} = 0.0275
Completeness to θ = 25.242°	99.9 %
Data / Restraints / Parameters	8965/0/427
Goodness-of-fit on F^2	1.036
Final *R* indexes [$I \geq 2\sigma(I)$]	R_1 = 0.0450 wR_2 = 0.1211
Final *R* indexes [all data]	R_1 = 0.0722 wR_2 = 0.1384
Largest peak/hole [$eÅ^{-3}$]	0.74/-0.55

4.2 Visualizations of the molecule structure

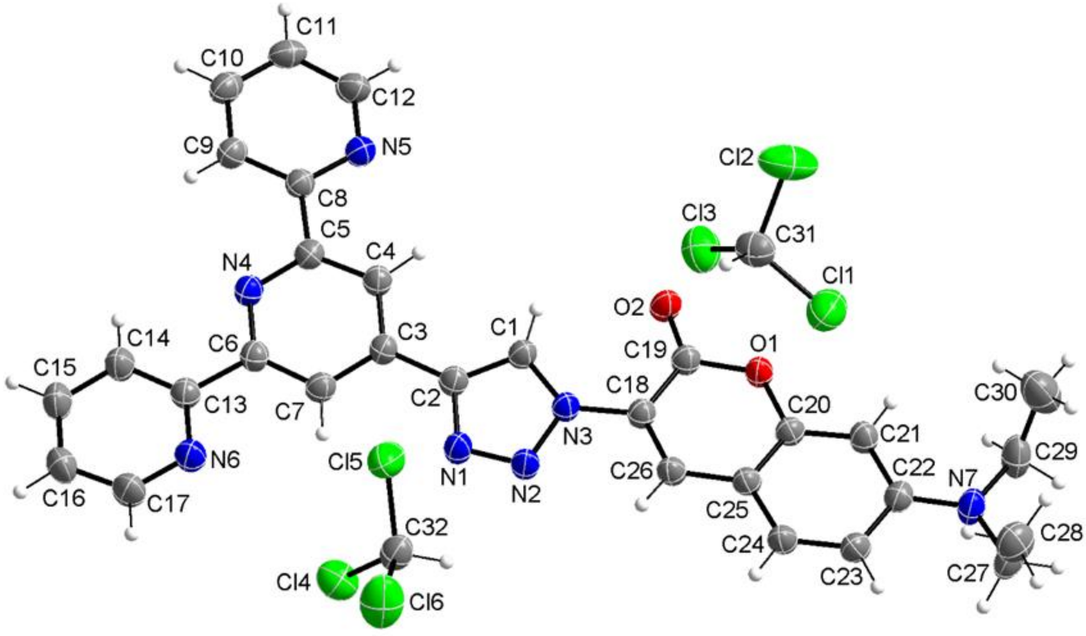

Figure S10. Molecular structure of **1** with atomic labels. Displacement ellipsoids are shown at the 50% probability level.

Table S2. Comparison of various fluorescent probes **Zn1-Zn6** for the detection of cations.

Fluorescent probe	$\lambda_{f(max)}$	metal	Limit of detection (LOD)	sensing mechanism	application
**Zn1**	486 nm (CH_3CN)	Zn^{2+}	-	Photoinduced Electron Transfer (PET)	Zn^{2+} imaging in HeLa cells
**Zn2**	444 nm 530 nm (DMSO/water) (v/v,1/99)	Zn^{2+}	-	Intramolecular Charge Transfer (ICT) (ratiometric)	Zn^{2+} imaging in HeLa cells
**Zn3**		Zn^{2+} and pyrophosphate(PPi)	-	Turn ON Fluorescent probe (Zn^{2+}) ratiometric (PPi)	in vivo imaging in *C.elegans* and in Hi-5 cells
**Zn4**	500 nm 609 nm (DMSO/water) (v/v,1/99)	Zn^{2+}	0.65 ppb	Intramolecular Charge Transfer (ICT) (ratiometric)	Zn^{2+} imaging in HeLa cells
**Zn5**	511 nm 562 nm (CH_2Cl_2)	Zn^{2+}	-	Intramolecular Charge Transfer (ICT) (ratiometric)	-
**Zn6**	513 nm 547 nm (CH_2Cl_2)	Zn^{2+}	-	Intramolecular Charge Transfer (ICT) (ratiometric)	-
**1**	485 nm (CH_3CN)	no cation selectivity	-	Intramolecular Charge Transfer (ICT) Fluorescence quenching	-
